# *Acinetobacter baumannii* Infection Inhibits Airway Eosinophilia and Lung Pathology in a Mouse Model of Allergic Asthma

**DOI:** 10.1371/journal.pone.0022004

**Published:** 2011-07-18

**Authors:** Hongyu Qiu, Rhonda KuoLee, Greg Harris, Hongyan Zhou, Harvey Miller, Girishchandra B. Patel, Wangxue Chen

**Affiliations:** 1 Institute for Biological Sciences, National Research Council Canada, Ottawa, Ontario, Canada; 2 Department of Biology, Brock University, St. Catharines, Ontario, Canada; University of Nebraska Medical Center, United States of America

## Abstract

Allergic asthma is a dysregulation of the immune system which leads to the development of Th2 responses to innocuous antigens (allergens). Some infections and microbial components can re-direct the immune response toward the Th1 response, or induce regulatory T cells to suppress the Th2 response, thereby inhibiting the development of allergic asthma. Since *Acinetobacter baumannii* infection can modulate lung cellular and cytokine responses, we studied the effect of *A. baumannii* in modulating airway eosinophilia in a mouse model of allergic asthma. Ovalbumin (OVA)-sensitized mice were treated with live *A. baumannii* or phosphate buffered saline (PBS), then intranasally challenged with OVA. Compared to PBS, *A. baumannii* treatment significantly reduced pulmonary Th2 cytokine and chemokine responses to OVA challenge. More importantly, the airway inflammation in *A. baumannii*-treated mice was strongly suppressed, as seen by the significant reduction of the proportion and the total number of eosinophils in the bronchoalveolar lavage fluid. In addition, *A. baumannii*-treated mice diminished lung mucus overproduction and pathology. However, *A. baumannii* treatment did not significantly alter systemic immune responses to OVA. Serum OVA-specific IgE, IgG1 and IgG2a levels were comparable between *A. baumannii*- and PBS-treated mice, and tracheobronchial lymph node cells from both treatment groups produced similar levels of Th1 and Th2 cytokines in response to *in vitro* OVA stimulation. Moreover, it appears that TLR-4 and IFN-γ were not directly involved in the *A. baumannii*-induced suppression of airway eosinophilia. Our results suggest that *A. baumannii* inhibits allergic airway inflammation by direct suppression of local pulmonary Th2 cytokine responses to the allergen.

## Introduction

Allergic asthma is a chronic, reversible airway inflammatory disease of significant public health importance. Although the exact mechanism is not clear, allergic asthma appears to result from allergen specific type 2 T helper (Th2) lymphocyte proliferation with concomitant excessive production of Th2 cytokines interleukin (IL)-4, IL-5, IL-13 and/or IL-25 [1]. The allergen-specific Th2-like immune responses include secretion of allergen specific IgE, overproduction of bone marrow eosinophils, airway eosinophilia, mucus secretion by goblet cells, and smooth muscle contraction, all collectively contributing to airway hyperreactivity [2], [3].

The gene-environment interaction seems to modulate the aberrant immune responses to allergens, which leads to the development and perpetuation of asthma [2]. In the last several decades, the incidence of asthma has increased rapidly in both developed and developing countries [4], with the estimated number of asthmatic patients increasing from about 130 million people in the mid-1990s to 330 million in 2008 [5], [6]. However, there are notable disparities in the prevalence of asthma between developed and developing countries, and between urban and rural areas of the same country [5]. It is postulated by the hygiene hypothesis that improved living conditions (such as better hygiene and reduced incidences of infectious diseases) in industrialized countries and urban areas may somewhat contribute to the development of allergic asthma [5], [7].

According to the hygiene hypothesis, neonatal and early childhood exposure to certain microbes and their products may shift the immune response toward a Th1 phenotype, or activate regulatory T cells and enhance IL-10 production, and thus suppress the aberrant allergen-specific Th2 responses and alleviate or inhibit the development of clinical symptoms of allergic asthma [8]–[11]. This idea is supported by many experimental and clinical studies with several microbes and their products [8]–[17].

*Acinetobacter baumannii* is a gram-negative, extracellular bacterium that causes nosocomial and community-acquired pneumonia and other infections [18]. Previous studies in our and other laboratories have shown that intranasal (i.n.) administration of *A. baumannii* induces acute bronchopneumonia characterized with neutrophil infiltration at the first 72 h after infection [19], [20], followed by macrophages and lymphocytes infiltration, and rapid clearance of the bacteria ∼4 days after infection. Although allergic asthma is primarily mediated by Th2-like immune responses, factors of the innate immune system can play important roles in disease initiation and progression. For example, as the ligand of TLR4, LPS co-administration with allergens was found to either inhibit or exacerbate the severity of asthmatic responses in mice [15]. Adoptive transfer of resident alveolar macrophages also inhibited the airway hyperresponsiveness to OVA challenge in rats [21]. Since *A. baumannii* lung infection significantly modulates the host innate immune response, we examined the effect of *A. baumannii* infection/treatment of ovalbumin (OVA)-sensitized mice on the development of airway eosinophilia and associated pulmonary pathology upon subsequent OVA challenge, using a mouse model of OVA-induced allergic asthma. Our results showed that *A. baumannii* infection suppressed both OVA-specific Th1 and Th2 cytokine responses and the expression of eotaxins in the lung, through a TLR-4 and IFN-γ-independent mechanism. More importantly, the infection suppressed airway eosinophilia and associated lung pathology. The results of this study emphasize the importance of infection-associated innate immune responses in the regulation of the development of allergic asthma.

## Materials and Methods

### Mice

Six- to 8-week-old female C57BL/6 mice were purchased from Charles River Laboratories (St. Constant, Quebec, Canada). Female B6.129S7-Ifng^tm1Ts^/J (IFN-γ^−/−^), C57BL/10ScNJ (TLR4^−/−^) and C57BL/10SnJ (TLR4^+/+^) mice of a similar age were purchased from Jackson Laboratories (Bar Harbor, ME, USA). Mice were housed under specific pathogen-free conditions in the Animal Resources, Institute for Biological Sciences, National Research Council Canada (Ottawa) and given free access to sterile water and ovalbumin (OVA)-free mouse chow. The mice were housed and used in accordance with the recommendations of the Canadian Council on Animal Care Guide to the Care and Use of Experimental Animals. This study and all animal care/use protocols were approved (ID # 2006.20 and 2007.15) by the Institute for Biological Sciences (National Research Council Canada) Animal Care Committee.

### Airway eosinophilia induction and *A. baumannii* inoculation

**Figure 1:**
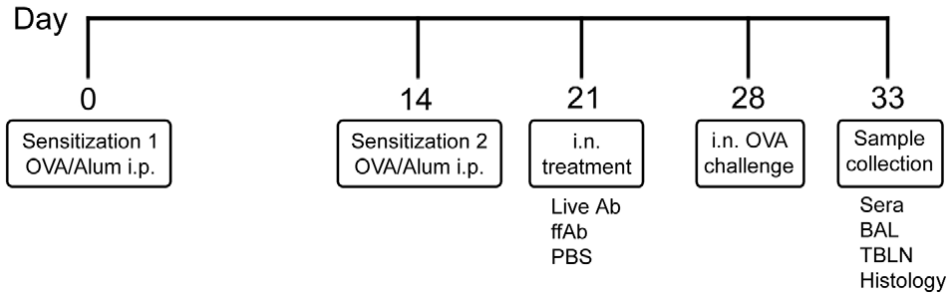
Experimental protocol for the study. Mice were sensitized by i.p. administration of 2 µg ovalbumin admixed with 100 µl alum at day 0 and 14. At day 21, the mice were treated by i.n. administration (50 µl volume) of PBS, live A. baumannii (∼10^8^ CFU) or formalin-fixed (ff) *A. baumannii* (∼10^8^ CFU). At day 28, mice were intranasally challenged with 100 µg OVA in 50 µl PBS or 50 µl PBS alone, as described in Material and Methods. Five days after challenge (day 33), mice were sacrificed for sample collection.

Airway eosinophilia in the mouse model was induced as described before [17] and is illustrated in Figure 1. Briefly, mice were sensitized by intraperitoneal (i.p) injection of 2 µg OVA (Sigma Chemical Co., St Louis, MO, USA) admixed in 100 µl alum (Pierce Laboratories, Rockford, IL, USA) at day 0 and 14. Seven days later (day 21), mice were treated by intranasal (i.n.) administration (50 µl volume) of phosphate-buffered saline (PBS), live *A. baumanii* (∼10^8^ CFU) in PBS, or formalin-fixed *A. baumannii* (ffAb) in PBS (∼10^8^ CFU). Seven days post-treatment (day 28), the mice were intranasally challenged with 100 µg OVA in 50 µl of PBS. Five days after OVA challenge (day 33), the mice were sacrificed and samples collected for various assays as indicated below. For live *A. baumannii* treatment, fresh inocula were prepared for each experiment from a frozen stock culture of *A. baumannii* (ATCC 17961, American Type Culture Collection, Manassas, VA) as described previously [20]. The actual treatment concentration in each experiment was determined by plating 10-fold serial dilutions on brain heart infusion (BHI) agar supplemented with 50 µg/ml streptomycin. Our previous studies showed that, at this infection dose (∼10^8^ CFU), *A. baumannii* is generally cleared from the lungs in 4 days [20]. For obtaining the formalin-fixed *A. baumannii* cells, the cells were fixed by overnight incubation at 37°C in 10% neutral buffered formalin while gently rotating the culture vessel, followed by 3× washing and centrifugal harvesting in PBS.

### Bronchoalveolar lavage (BAL) and histopathology

Mice were sacrificed five days after i.n. OVA challenge. Sera were separated and stored at −80°C for antibody assays. The lungs were lavaged five times with 1 ml PBS supplemented with 3 mM EDTA and 1% fetal bovine serum [20]. Total lavage cell numbers were enumerated using a haemocytometer, and differential cell counts were determined on cytospin preparations stained with Hema-3 stain set (Fisher Scientific, Middletown, VA, USA). BAL fluid was centrifuged at 3,000*×g* for 7 min, and supernatants were collected and stored at −80°C. In some experiments, the lungs were removed immediately after lavage and immersed in 10% neutral buffered formalin. The tissues were processed by standard paraffin embedding methods (Department of Pathology and Laboratory Medicine, University of Ottawa, Ottawa, Canada), sectioned (4 µm thick), and stained with haematoxylin and eosin (H&E) or periodic acid-Schiff (PAS) for histopathological evaluation. In some experiments, the lungs from each mouse were minced into small pieces using scissors, and homogenized in 2 ml of saline supplemented with Complete® protease inhibitors (Roche Diagnostics Canada, Laval, QC, Canada). The supernatant was collected by centrifugation (3,000*×g* for 7 min) and stored at −80°C until used for cytokine assay.

Total RNA isolation and real-time reverse transcriptase polymerase chain reaction (qRT-PCR) for cytokine mRNA expression

For RNA extraction, the lungs were immersed immediately in RNAlater® (Qiagen Inc., Valencia CA) and stored at −20°C. Total RNA was extracted using TRIzol (Invitrogen Canada, Burlington). Relative abundance of cytokine mRNA in lungs were evaluated using a real-time RT-PCR-based method, as described elsewhere [17]. Briefly, single stranded cDNA was prepared through reverse transcription, cytokine genes were amplified and quantified using primers and probes designed with the Primer3 program [22]. Levels of PCR products were normalized to the housekeeping gene β-2-microglobulin (β2m), and data presented as the average of relative expression values in *A. baumannii*-treated mice compared with those in the corresponding tissues of PBS-treated mice [17].

### *In vitro* re-stimulation culture of tracheobronchial lymph node (TBLN) cells

To assess *in vitro* cytokine responses to OVA stimulation, TBLNs were collected for the preparation of single-cell suspensions (4×10^6^ cells/ml). TBLN cells were cultured in 48-well tissue culture plates in RPMI-1640 (Sigma-Aldrich, St Louis, MO, USA) supplemented with 10% heat-inactivated fetal bovine serum (Invitrogen, Grand Island, NY, USA), 50 µM 2-mercaptoethanol (Sigma-Aldrich), and 100 U/ml penicillin G and 100 µg/ml streptomycin (Invitrogen). The cells were either stimulated with 1 mg/ml OVA or medium alone. Culture supernatants were collected 72 h later (500*×g* centrifugation for 10 min) and stored at −80°C for cytokine assays.

### Cytokine and chemokine assays

The levels of eotaxin-1, IFN-γ, IL-4, IL-5, IL-10, IL-12p40, and IL-13 in the BAL fluid and lung homogenate supernatants were measured by the Milliplex mouse cytokine/chemokine kit (Millipore, Billerica, MA) on a Luminex® 100IS system (Luminex Corp, Austin, TX, USA) [23]. The analysis was done in duplicate, and the cytokine concentrations were calculated against the standards using Beadview® software (ver. 1.03, Upstate). The detection limit was <2 pg/ml for IL-4, IL-10, and IL-13, 5.54 pg/mL for IL-12p40, 8.97 pg/ml for IFN-γ, and 30.9 pg/mL for IL-5, respectively. IL-17A levels in the BAL fluid and lung homogenates were determined by mouse IL-17A ELISA Ready-Set-Go kit (eBioscience, San Diego, CA, USA), and the limit of detection was 6 pg/ml.

### Enzyme-linked immunosorbent assay (ELISA) for ovalbumin-specific immunoglobulin isotype

Serum OVA-specific IgE were assayed using anti-IgE mAb- (4B-39, BD Biosciences, Mississauga, Ontario, Canada) coated microtiter plates, and detected by an ELISA assay using OVA-biotin/streptavidin-horseradish peroxidase (HRP) in conjunction with TMB substrate (KPL Inc., Gaithersburg, MD, USA). Serum OVA-specific IgG1 and IgG2a were measured using OVA-coated microtiter plates (Immulon 2, Thermo Labsystems, Franklin, MA, USA) and detected using alkaline phosphatase conjugated goat anti-mouse IgG1 or IgG2a, respectively (Caltag Laboratories, Burlingame, CA, USA), in conjunction with pNPP substrate (KPL Inc.) [24].

### FACS analysis of regulatory T (Treg) cells

The percentages of Treg (CD4^+^CD25^+^Foxp3^+^) cells in the BAL fluid were determined by FACS analysis using the One Step Staining Mouse Treg Flow™ Kit (BioLegend, San Diego, CA). Briefly, BAL cell samples were washed in PBS containing 1% BSA. Aliquots containing ∼10^6^ cells were permeabilized using fixation/permeabilization buffer, following the manufacturer's protocol, and then stained using Alexa Fluor® 488 anti-mouse FOXP3/CD25 PE/CD4 PerCP antibody cocktail or 20 µl Alexa Fluor® 488 rat IgG2b, κ isotype control/CD25 PE/CD4 PerCP antibody cocktail for 30 min at 4°C. After staining, the cells were washed twice with the above PBS solution, and analyzed by FACS Canto II flow cytometer (BD Biosciences, San Jose, CA) using FACS Diva (BD Biosciences, San Jose, CA) software.

### Statistical analyses

All parametric data are presented as mean ± standard deviation (SD) for each group. Differences between groups were analyzed by Student's t-test or by one-way and two-way ANOVA followed by the Bonferroni multiple comparison test, when appropriate. P<0.05 was considered to be statistically significant. All statistical analyses were done using GraphPad Prism software (version 4.0, GraphPad Software, San Diego, CA, USA).

## Results

### *Acinetobacter baumannii* infection inhibits airway eosinophilia and associated pulmonary pathology in OVA-sensitized/challenged mice

**Figure 2:**
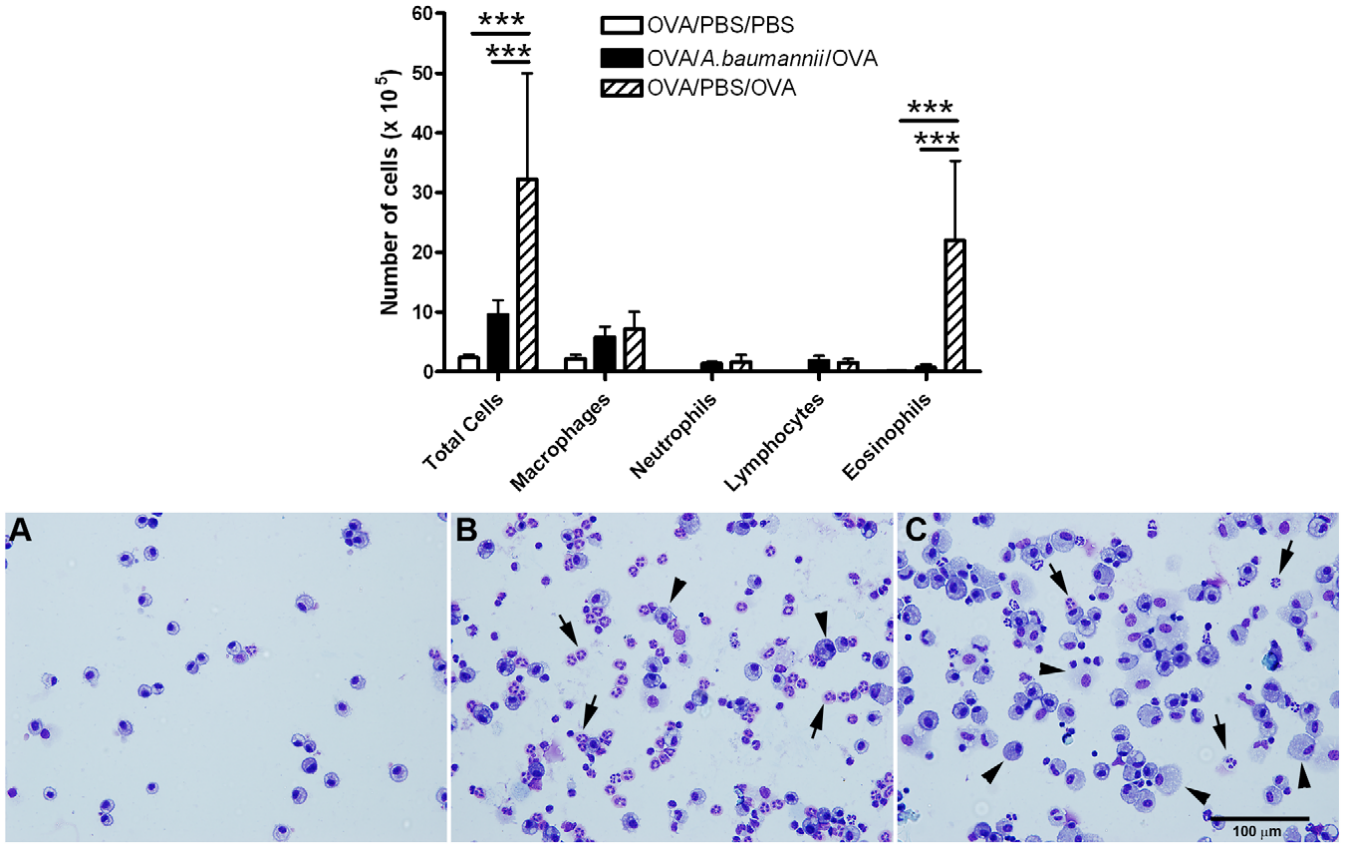
Inhibition of airway eosinophilia in OVA-sensitized mice by live *A. baumannii*. Mice were sensitized by i.p. administration of OVA/alum on days 0 and 14, then treated with *A. baumannii* or PBS on day 21, and intranasally challenged with OVA on day 28, as described in Fig. 1. Five days after the i.n. OVA challenge, mice were euthanized, and their lungs were lavaged. Upper panel: Total and differential cell counts in the bronchoalveolar lavage (BAL) fluid were enumerated on cytospin preparations. Each bar represents the mean total number of respective types of cells in the BAL fluid ± SD (n = 5). The data presented represent 1 of at least 2 separate experiments with similar results. ***P<0.001. Lower panel: The BAL cells from OVA-sensitized, PBS-challenged mice (A) consist of predominantly alveolar macrophages whereas the BAL cells from PBS-treated, OVA-challenged mice (B) consist of mainly eosinophils (arrows) with a donut- or horseshoe-shaped nucleus and a pink granular cytoplasm. The majority of BAL cells from *A. baumannii*-treated, OVA-challenged mice (C) are large alveolar macrophages (arrowheads) with a foamy cytoplasm. HemaStat-3 staining, bar = 100 µm.

To examine the effect of *A. baumannii* infection on the development of airway eosinophilia and associated pulmonary pathology, groups of OVA-sensitized C57BL/6 mice were intranasally inoculated with *A. baumannii* (*A. baumannii* treatment) or PBS (PBS treatment) 3 weeks after sensitization. BAL fluid from OVA-sensitized mice that had been treated with PBS prior to challenging with OVA (OVA/PBS/OVA), had infiltration of large numbers of eosinophils and mononuclear cells, as well as some neutrophils (Figure 2). In contrast, i.n. inoculation of OVA-sensitized mice with live *A. baumannii* prior to challenge with OVA (OVA/*A. baumannii*/OVA) significantly inhibited eosinophil influx into the bronchoalveolar space (P<0.001), without significantly affecting the recruitment of the other cell types (neutrophils, macrophages or lymphocytes), as compared with PBS-treated mice (Figure 2). As expected, the BAL cells from OVA-sensitized and i.n. PBS-challenged mice (OVA/PBS/PBS) contained predominantly (99%) alveolar macrophages (negative control).

**Figure 3:**
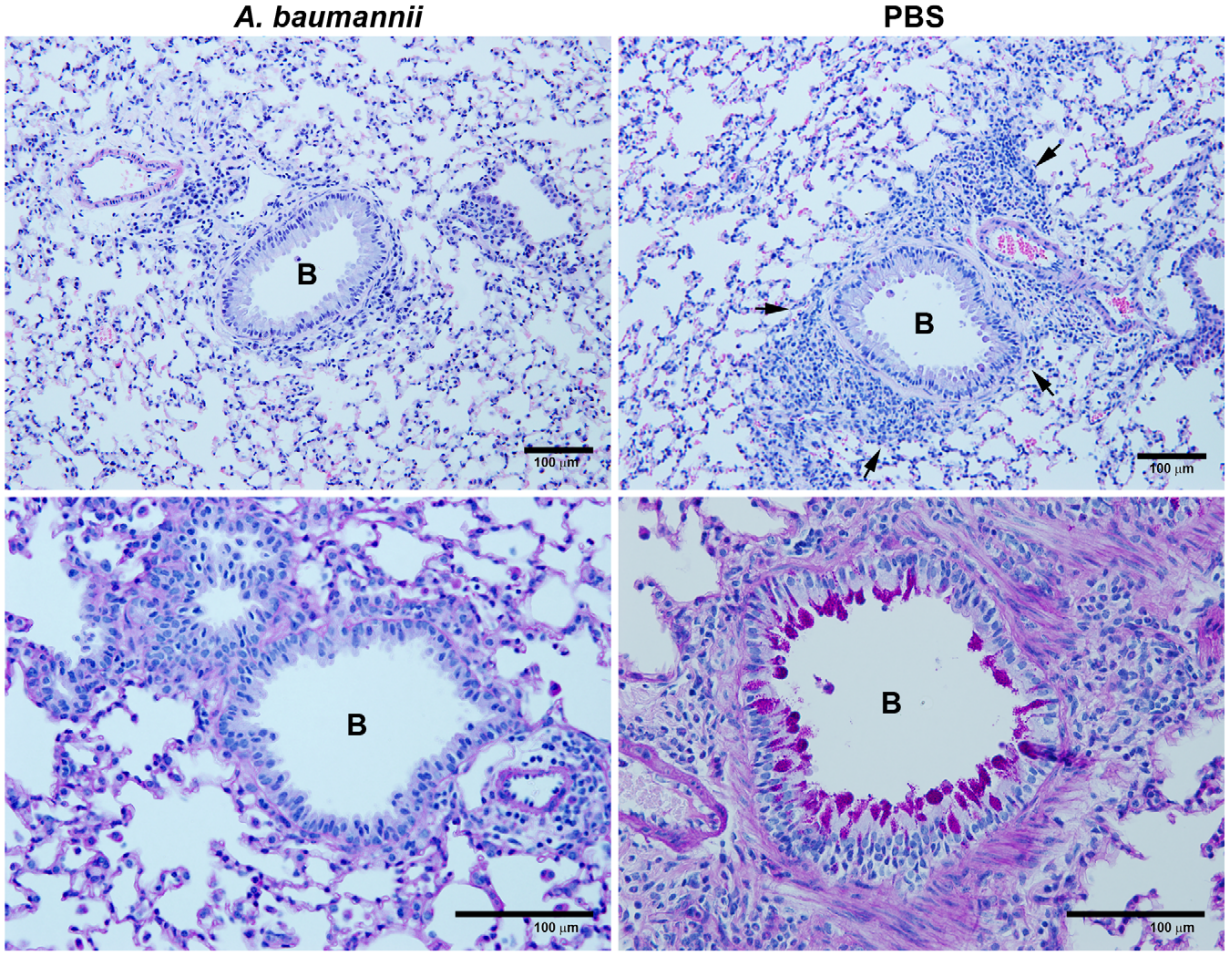
Representative lung histopathology from OVA-sensitized mice treated with *A. baumannii*. The mice were sensitized and treated as described in Figure 2 and killed 5 days after the OVA challenge. Note the severe pulmonary inflammation in the areas adjacent to various sized airways in PBS-treated, OVA-sensitized/challenged mouse (arrows, top right panel) and the presence of large numbers of mucus-producing goblet cells (dark purple) (bottom right panel) whereas the inflammation, goblet cell hyperplasia and mucus production were relatively minor in the lungs of mice treated with *A. baumannii* (top left and bottom left panels). B = bronchus. Top panels stained with H&E; bottom panels stained with periodic acid-Schiff, Bar = 100 µm.

We also examined the histopathogical changes in the lungs of these mice. As shown in Figure 3, i.n. OVA challenge of the sensitized mice treated with PBS (right panel) induced significant perivascular and peribronchial infiltration of various types of inflammatory cells, especially eosinophils, whereas OVA challenge of *A. baumannii*-treated, sensitized mice substantially suppressed inflammatory cell infiltration and associated pulmonary pathology (left panel). Excess mucus secretion is an important pathophysiological indicator of allergic asthma. Compared with PBS-treated mice, the mice treated with *A. baumannii* had substantially reduced mucus production by bronchial epithelial cells (Figure 3). These results indicated that infection with *A. baumannii* significantly inhibited the development of allergic airway eosinophilia and associated pulmonary pathology in mice.

**Figure 4:**
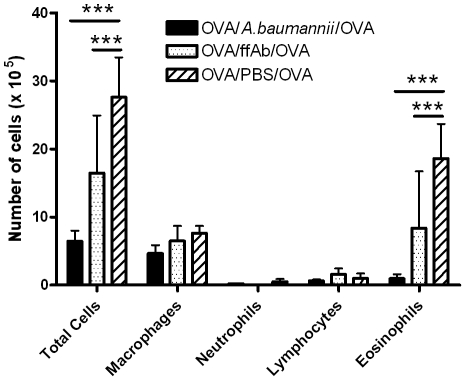
Inhibition of airway eosinophilia in OVA-sensitized mice by intranasal treatment with live or formalin-fixed *A. baumannii* (ffAb). Mice were sensitized by i.p. administration with OVA/alum on days 0 and 14. Sensitized mice were i.n. treated with either 1×10^8^ CFU live or formalin-fixed *A. baumannii* on day 21 and i.n. challenged with OVA on day 28. Cells in the bronchoalveolar lavage (BAL) fluid were collected 5 days after OVA challenge and differential cell types were enumerated on cytospin preparations. Each bar represents the mean total numbers of respective types of cells in the BAL fluid ± SD (n = 5). The data presented represent 1 of at least 2 separate experiments with similar results. ***P<0.001.

Several groups, including us, have previously shown that certain crude or purified bacterial components (such as lipopolysaccharide, LPS) can suppress allergic airway eosinophilia as effectively as the live bacterial infection [9], [14], [15], [17], [25], [26]. To examine this possibility, OVA-sensitized mice were treated by i.n. administration of formalin-fixed *A. baumannii* (ffAb) before OVA challenge, and the eosinophil responses were compared with those in mice intranasally treated with PBS or live *A. baumannii*. Compared to the BAL fluid from the PBS treatment group, BAL fluid from the ffAb treatment group had ∼50% lower eosinophil infiltration upon i.n. OVA challenge (Figure 4). However, the live *A. baumannii* infection suppressed pulmonary eosinophil responses by more than 90%, compared to the PBS control treatment. These observations indicate that although certain component(s) of *A. baumannii* can partially suppress allergic airway inflammation, active infection is necessary to maximize the inhibitory effect.

### *A. baumannii* infection does not significantly alter serum OVA-specific IgE and IgG subclass levels

**Figure 5:**
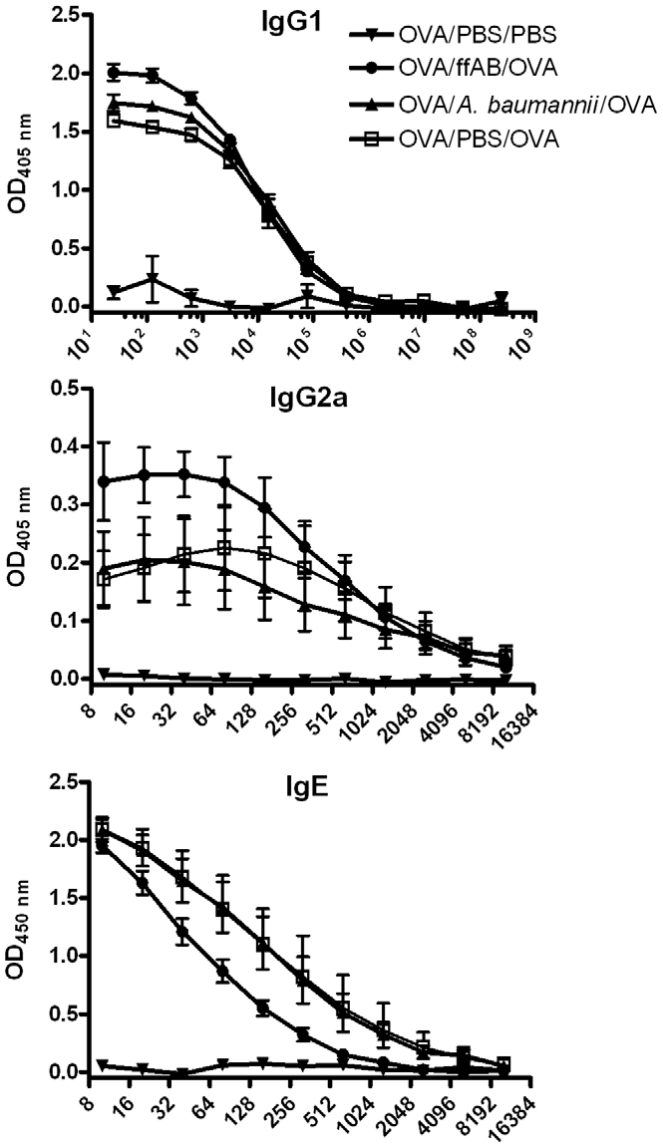
OVA-specific serum IgG1, IgG2a, and IgE levels in OVA-sensitized mice. Mice were OVA-sensitized as described in Figure 2 and treated with PBS and challenged with PBS (OVA/PBS/PBS),,treated with *A. baumannii* and challenged with OVA (OVA/*A. baumannii*/OVA), treated with formalin-fixed *A. baumanii* and challenged with OVA (OVA/ffAb/OVA), or treated with PBS and challenged with OVA (OVA/PBS/OVA). Groups of 5 mice were euthanized five days after i.n. OVA challenge, and serum was collected. The OVA-specific IgG1, IgG2a and IgE levels were measured using ELISA. Each data point represents the mean OD value ± SD of five mice in each group.

Allergic asthma is generally recognized as a Th2-dependent immune response with increased serum antigen-specific IgE and IgG1 production [1], [2]. As a first step to address the potential mechanism of *A. baumannii*-induced inhibition of airway eosinophilia, we examined the effect of i.n. treatment with live or formalin-fixed *A. baumannii* on the changes in the serum IgE and IgG subclasses (IgG1 and IgG2a). In agreement with published reports [8], [12], [14], [27], simply sensitizing mice with OVA-alum at 0 and 14 d (OVA/PBS/PBS group) induced only marginal levels of OVA-specific IgE, IgG1 and IgG2a antibodies (Figure 5). As expected, intranasal OVA challenge of the sensitized mice (OVA/PBS/OVA) induced a remarkable increase in serum OVA-specific IgG1, IgG2a, and IgE in sensitized mice. However, treatment of sensitized mice with live or formalin-fixed *A. baumannii* showed no significant effect on the serum OVA-specific IgE or IgG1 levels as compared with PBS treatment group (Figure 5). The *A. baumannii* treatment also showed no effect on the Th1-dependent OVA-specific IgG2a levels (Figure 5).

### Effect of i.n *A. baumannii* treatment on lung cytokine/chemokine responses in allergic mice

**Figure 6:**
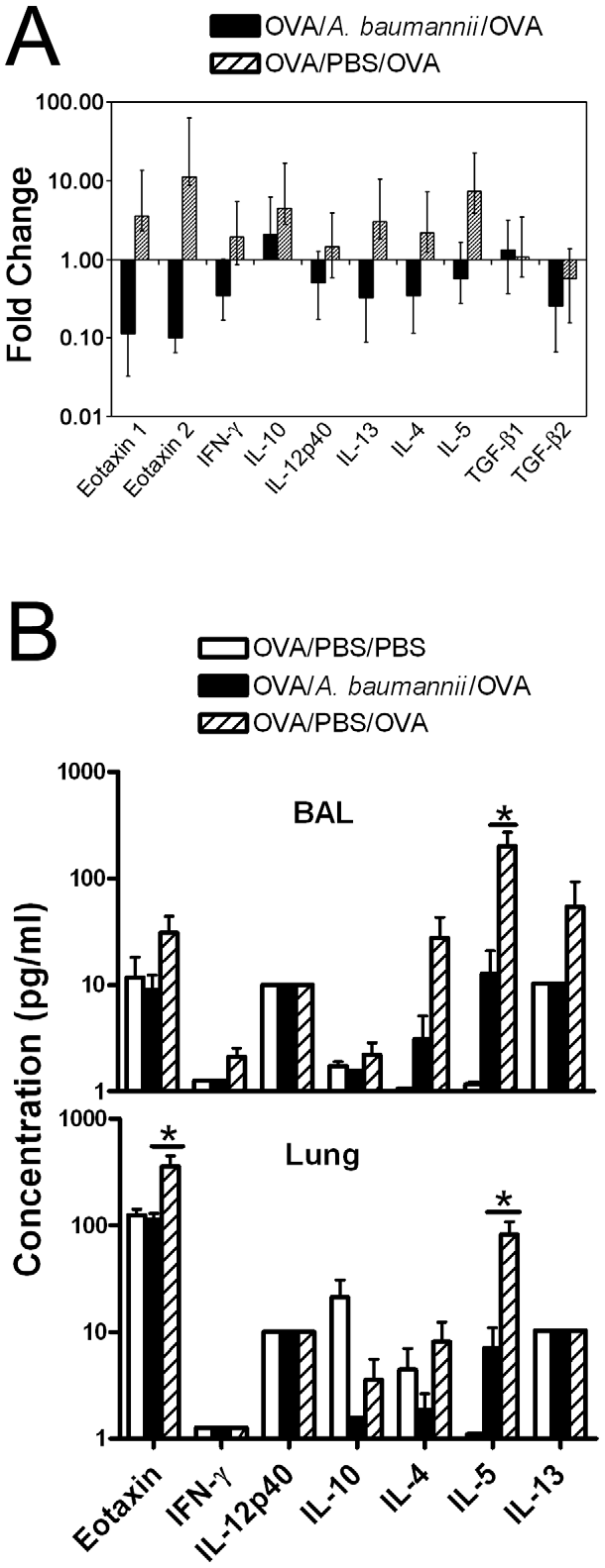
Cytokine responses in the lung and BAL fluid of OVA-sensitized mice following OVA challenge. (A) Real-time PCR analysis of cytokine mRNA expression in lung tissues in OVA-sensitized and *A. baumannii* treated mice following i.n. OVA challenge. Mice were sensitized and treated as described in Figure 2 and were euthanized 5 days after i.n. OVA challenge. The lungs were collected for RNA extraction. Relative levels of cytokine mRNA expression were determined by real-time RT-PCR analysis as described in Materials and methods. Mouse β-2 microglobulin RNA was measured and used to calculate relative expression using the formula Rel Exp = 2^−(ΔΔCT)^. Results shown are the average and ranges (error bars) of relative expression values determined using cDNA from *A. baumannii*- or PBS-treated, OVA challenged mice in relation to the corresponding expression levels in PBS challenged mice (n = 5 for all groups). (B) Effect of *A. baumannii* infection on cytokine levels in BAL fluid and lung homogenates in OVA-sensitized mice following i.n. OVA challenge. Mice were sensitized and treated as described in Figure 2 and were euthanized 5 days after i.n. OVA challenge. The levels of indicated cytokines in BAL fluid and in the lung homogenate supernatants were measured on a Luminex 100IS system using the Milliplex MAP mouse cytokine/chemokine detection kit (Millipore). Each bar represents the mean pg cytokine/mL ± SD (n = 5). The data are representative of two to three independent experiments. *P<0.05 compared to the PBS-treated group.

It is well recognized that allergic airway eosinophilia and inflammatory responses are regulated by multiple chemokines [28]–[31] and Th2 cytokines [32]–[34]. To examine the effect of *A. baumannii* on the cytokine/chemokine responses to OVA challenge in OVA-sensitized mice, the mRNA expression of cytokine IFN-γ and IL-12p40 (Th1 associated), IL-4, IL-5, and IL-13 (Th2-associated), and IL-10 (regulatory/suppressor function associated) in the lungs, as well as their corresponding protein levels in the BAL fluid and in lung homogenate supernatants were analyzed from mice killed at day 5 after i.n OVA challenge. Compared with PBS treatment, live *A. baumannii* treatment resulted in a substantial reduction in IL-4, IL-5 and IL-13 mRNA expression, as well as decreases in the mRNA expression of IFN-γ, IL-10, and IL-12p40 (Figure 6A). Consistent with the mRNA expression patterns, the protein levels of these cytokines were also generally lower in the lungs and BAL fluids of *A. baumannii*-treated mice as compared to the PBS-treated mice, although these differences were only statistically significant for IL-5 (P<0.05)(Figure 6B).

We also found that the mRNA expression of eotaxin 1 and eotaxin 2, the key chemokines in the induction of eosinophil influx into allergic tissue in mice and humans [28], [31], [32], [35], was markedly reduced in *A. baumannii*-infected mice, compared to the high expression levels after OVA challenge in PBS-treated mice (Figure 6A). Correspondingly, the level of eotaxin-1 in the lung homogenates and, to a lesser degree in the BAL fluid was significantly lower (P<0.05) in *A. baumannii*-infected mice (Figure 6B).

**Figure 7:**
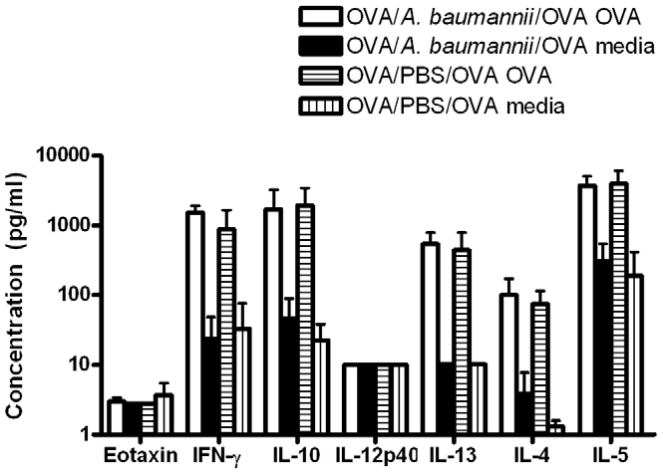
Cytokine responses to *in vitro* re-stimulation of tracheobronchial lymph node (TBLN) cells from *A. baumannii*-treated mice. Groups of OVA-sensitized C57BL/6 mice were i.n. treated with either *A. baumannii* or PBS 7 days before i.n. OVA challenge. Mice were killed 5 days after the challenge and their TBLNs were collected and used for *in vitro* culture to determine cytokine production in response to OVA stimulation. Single cell suspensions (4×10^6^ cells/mL) were re-stimulated *in vitro* for 48 h with either OVA (1 mg/mL) or culture medium only. The cytokine levels in the supernatants were determined by Luminex. Data are presented as mean concentration (pg/ml) ± SD (n = 5), and are representative of two independent experiments.

To further examine the potential mechanism underlying the *A. baumannii*-induced suppression of allergic airway eosinophilia, we compared the cytokine responses to *in vitro* OVA re-stimulation of TBLN cells obtained from PBS- or *A. baumannii*-treated, OVA sensitized mice 5 days after an i.n. OVA challenge. As can be seen in Figure 7, similar to the TBLN cells from PBS-treated mice, the TBLN cells from *A. baumannii*-treated mice produced comparable amounts of IFN-γ, IL-10, IL-4, IL-5 and IL-13 in response to *in vitro* OVA re-stimulation. The levels of IL-12p40 and eotaxin were at the lower limit of detection. As expected, stimulation of TBLN cells from both groups of mice with medium alone induced only minimal amounts of cytokine production (Figure 7).

### TLR4 or IFN-γ is dispensable for *A. baumannii*-induced inhibition of airway eosinophilia

**Figure 8:**
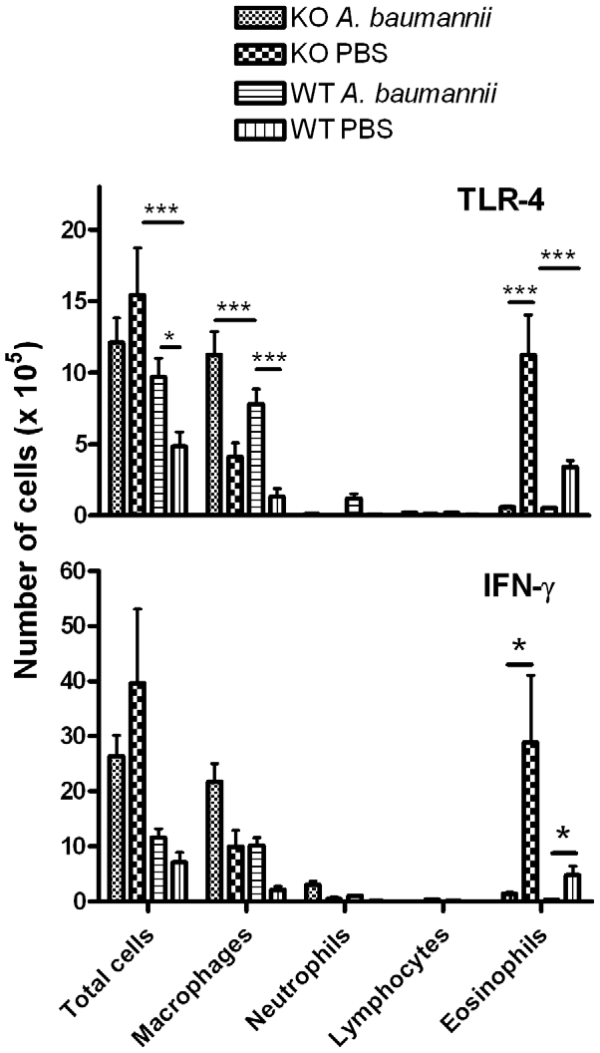
*A. baumannii* infection inhibits airway eosinophilia in OVA-challenged TLR-4−/− and IFN-γ−/− mice. Knock out (KO) and corresponding wild-type (WT) mice were sensitized i.p. with OVA/alum on days 0 and 14 and treated with live *A. baumannii* or PBS as described in Fig. 2. Mice were i.n. challenged with OVA on day 28. Cells in the bronchoalveolar lavage (BAL) fluid were collected 5 days after OVA challenge and different cell types were enumerated on cytospin preparations. Each bar represents the mean total number of respective types of cells per mouse lung ± SD (n = 5). *P<0.05 and ***P<0.001.

Since *A. baumannii* is a gram-negative bacterium that contains abundant amounts of LPS [36], we examined the potential contribution of LPS in the *A. baumannii*-induced inhibition of allergic airway eosinophilia, using TLR4−/− mice. In agreement with previous observations [15], [37], [38], OVA-sensitized TLR4−/− mice showed significantly higher airway eosinophil responses to i.n OVA challenge than did the OVA-sensitized wild type mice, suggesting that TLR4 signaling *per se* can efficiently suppress OVA-induced airway inflammation. However, *A. baumannii* infection significantly inhibited, and at a similar magnitude, the airway eosinophilia in both strains of mice (Figure 8). This result indicates that *A. baumannii*-induced suppression of allergic airway eosinophilia is independent of the TLR4 signaling pathway.

IFN-γ is an important Th1 cytokine that down-regulates Th2 cytokine responses, and it has been implicated in the development of allergic asthma [39]–[41]. We examined whether IFN-γ plays a role in *A. baumannii*-induced suppression of airway eosinophilia, using IFN-γ−/− mice. In comparison with PBS-treated wild type mice, the PBS-treated IFN-γ−/− mice displayed much stronger airway eosinophilia following i.n. OVA challenge, suggesting a general role for IFN-γ in the suppression of the immune responses to OVA challenge in OVA-sensitized mice (Figure 8). However, airway eosinophilia in both IFN-γ−/− and wild type mice was largely suppressed at a comparable magnitude after *A. baumannii* treatment (Figure 8), suggesting that *A. baumannii*-induced suppression of airway eosinophilia is not mediated by IFN-γ.

### Treg cells do not appear to mediate *A. baumannii*-induced inhibition of airway eosinophilia

**Figure 9:**
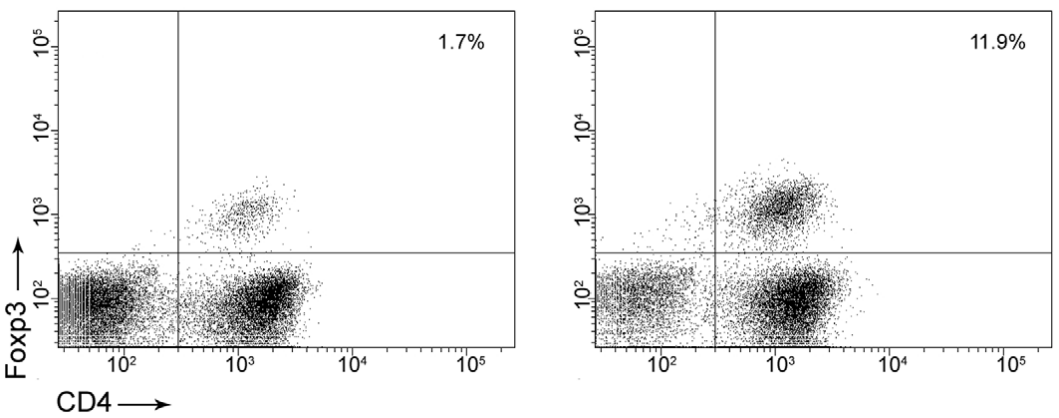
Regulatory T cells are not increased in BAL fluid after *A. baumannii* treatment of OVA-sensitized mice. BAL from *A. baumannii*-treated and PBS-treated, allergic mice were collected at 5 days after i.n. OVA challenge and the percentage of regulatory T cells was calculated by flow cytometry as determined by CD4^+^CD25^+^Foxp3^+^ staining. A representative dot plot from an *A. baumannii*-treated (left panel) and a sham-treated (right panel) mouse illustrates the percentage of CD4^+^Foxp3^+^ Treg cells gated on total lymphocytes in the BAL fluid.

Microbes can suppress the Th2 response of allergic airway eosinophilia through an elevated regulatory T cell response [42], [43]. We compared the number of Treg cells in BAL samples by flow cytometry and found that although there was a more remarkable increase in total BAL lymphocyte number in *A. baumannii*-treated, allergic mice than sham-treated, allergic mice, the percentage of Treg cells (defined as CD4^+^CD25^+^Foxp3^+^) was in fact lower in the *A. baumannii*-treated, allergic mice, than in sham-treated, allergic mice (1.7% vs 11.9%, Figure 9). In addition, the total number of Treg cells in the BAL of *A. baumannii*-treated, allergic mice (1.1×10^4^ Treg cells) was actually lower than the number of Treg cells in sham-treated, allergic mice (2.0×10^4^ Treg cells) (data not shown), suggesting that *A. baumannii*-induced suppression of airway eosinophilia is unlikely to be mediated through Treg cell-associated suppression of Th2 responses.

## Discussion

According to the hygiene hypothesis, exposure to microbes in early childhood and throughout the life may modulate immune responses during allergen stimulation, and prevent the development of allergic asthma. Based on this theory, several clinical and experimental studies that employed bacteria or bacterial component(s) to prevent or treat asthma have shown some promising results [9], while the mechanisms for such suppression remain largely undefined. For example, *Mycobacteria* spp., the predominantly studied bacteria in experimental and clinical treatment of allergic asthma [8], [12], [14], [42], may alleviate airway inflammation by inducing different immune responses to allergens. Intranasal administration of live BCG before OVA challenge suppressed IL-5 production and airway eosinophilia in an IFN-γ dependent manner [8], while subcutaneous treatment with killed *M. vaccae* before OVA sensitization inhibited allergic airway inflammation through the induction of a CD4^+^CD45RB^lo^ regulatory T cell secreted IL-10 and TGF-β [42]. Similarly, T cell-derived IL-10 has been found to be necessary to alleviate asthmatic symptoms when dead mycobacteria or lipoglycan are administered before OVA challenge [14]. Thus, bacterial suppression of allergic airway inflammation and eosinophila may depend on the specific timing of the administration, the route of administration, and the nature of the microbes themselves. Microbes can modulate the allergic airway eosinophilia by switching a predominantly Th2 phenotype to a predominantly Th1 phenotype, or by suppressing the Th2 responses through elevated regulatory T cell response.

*Acinetobacter baumannii* is a ubiquitous Gram-negative, opportunistic pathogen that frequently induces both nosocomial and community-acquired infections such as pneumonia, skin infection, urinary tract infection and bacteremia [44]–[46]. *A. baumannii* infection has recently emerged as a major cause of nosocomial infections worldwide, likely because the bacteria can survive on the surface of medical devices such as catheters and ventalitors, as well as on the hands of hospital staff for extended periods of time, and can easily spread through water droplets in the air [18], [46]. Moreover, *A. baumannii* infections have become increasingly difficult to treat because of the bacteria's rapid development of resistance to multiple antibiotics [18], [47]. In this study, we found that i.n. administration of live, and to a lesser extent, formalin fixed *A. baumannii* significantly inhibited airway eosinophilia in mice. Live *A. baumannii* treatment largely suppressed pulmonary Th2 cytokines IL-4, IL-5 and IL-13 production, as well as eosinophil-chemotactic chemokine eotaxin 1 and eotaxin 2. However, levels of Th1 cytokines IL-12 and IFN-γ in the lung (BAL fluid and tissue homogenate supernatant) were not significantly altered. Moreover, TBLN cells from *A. baumannii* - and PBS-treated mice secreted similar amounts of cytokines upon in *in vitro* stimulation, and the levels of serum OVA-specific IgE, IgG1 and IgG2a were comparable. These results imply that *A. baumannii* infection did not change systemic immune responses to OVA from a Th2 to Th1 type, which is in contrast to several previous studies with other bacterial species such as *Chlamydia spp*, [48], *Listeria monocytogenesis* [10], and the live vaccine strain of *Francisella tularensis* (LVS) [17]. Since *A. baumannii* is an extracellular bacterium, it is likely that it may stimulate host immune responses that are different than those induced by the intracellular bacteria mentioned above. Indeed, several extracellular bacteria such as *Streptococcus pneumoniae* and *Bordetella pertussis* alleviate airway eosinophilia [25], [26]. Compared to saline treatment, treatment of mice with *S. pneumoniae* after OVA sensitization resulted in significant reduction of OVA-specific Th2 cytokines IL-5 and IL-13 responses by their TBLN cells without significant increases in IFN-γ production [25]. Similarly, administration of heat-killed whole cell *B. pertussis* during sensitization and before OVA challenge also significantly suppressed airway eosinophilia and lung inflammation, which correlated with suppressed Th2 cytokine (IL-4 or IL-5) responses without the increased Th1 cytokine (IL-12 or IFN-γ) levels [26]. It is not clear, however, whether administration of this bacterium changed the systemic immune responses. Similar to these extracellular bacteria, *A. baumannii*-induced inhibition of airway eosinophilia is associated with suppressed airway Th2 cytokine and chemokine production, without the enhancement of Th1 responses. However, in contrast to *S. pneumoniae*, *A. baumannii* treatment did not change the host systemic immune responses to OVA. This is probably due to the fact that pulmonary *A. baumannii* infection is acute and largely limited to the lungs, and the infection may not be extensive and persistent enough to modulate the systemic immune responses to OVA. Moreover, using IFN-γ−/− mice, we demonstrated that IFN-γ is not essential for *A. baumannii* induced suppression of allergic inflammation (Figure 8). The expression of IL-10 in lungs or upon *in vitro* OVA stimulation of TBLN lymphocytes from *A. baumannii*-treated mice also showed no differences in comparison to those of PBS-treated control mice after OVA challenge. Real-time RT-PCR results also indicated that pulmonary TGF-β production was similar between PBS- or *A. baumannii*-treated mice. In addition, we have demonstrated that *A. baumannii*-treated allergic mice appear to have fewer, rather than more, Treg cells in their BAL than do PBS-treated allergic mice (Figure 9). These results suggest that regulatory T cells may not be involved in the inhibition of allergic response by *A. baumannii* treatment. These findings cumulatively suggest that *A. baumannii*-induced inhibition of airway eosinophilia and associated pulmonary pathological changes did not involve the systemic suppression of Th2 associated factors or the elevation of Th1-specific parameters.

*A. baumannii*-induced alleviation of allergic airway inflammation is not an isolated observation in the *Acinetobacter* species, since it has been previously shown that i.n. treatment of mice with *A. lwoffii* F78, a non-pathogenic *Acinetobacter* isolate cultured from farm cowsheds, also suppressed airway eosinophilia and airway hyperresponsiveness [49]. However, unlike *A. baumannii*, *A. lwoffii* activates dendritic cells with increased expression of surface activation markers CD40, CD80, CD86 and MHCII, and induces highly Th1 polarizing immune responses including the enhanced expression of delta-4 mRNA and increased secretion of IL-12 secretion, as well as reduced mRNA expression for Jagged-1 [49]. However, there is no information on whether the inhibition is correlated with serum antibody levels/changes, or IL-4, 5, 13, and IFN-γ production. In our work, we used a clinically relevant *Acinetobacter* species, and showed similar suppression of the allergic airway responses. Moreover, our study indicates that alleviated airway inflammation in *A. baumannii*-treated mice is associated with suppressed Th2 cytokine and chemokine eotaxin expression in the lung. However, it is not entirely clear if *A. lwoffii* and *A. baumannii* utilize fundamentally different immunoregulation mechanisms in the inhibition of allergic airway eosinophilia, since the treatment regime used in *A. lwoffii* studies was quite different from the present study in that *A. lwoffii* treatment started 10 days before antigen sensitization and continued every second day throughout the whole sensitization and challenge phases of the study [49].

Our study showed that active infection with *A. baumannii* is not essential for the inhibition of allergic airway eosinophilia, and killed whole cells (formalin-fixed) are also capable of inhibiting the development of airway eosinophila, although to a lesser extent (Figure 4). *A. baumannii* is a gram-negative bacterium containing abundant LPS, and recent studies have shown that earlier childhood exposure to bacterial LPS is correlated with reduced risk of atopy such as hay fever and allergic airway diseases [50]. Treatment of mice with *Escherichia coli* LPS suppresses the development of airway hyperreactivity in mouse models of asthma [15], [37], [38]. Since LPS has been implicated in the regulation of allergic asthma development mainly through TLR-4 signalling pathway [51], we studied the effect of *A. baumannii* administration on allergic responses in TLR4−/− mice. Although PBS-treated TLR-4−/− mice showed stronger airway eosinophilia than PBS-treated wild type mice, indicating suppression of airway inflammation by TLR-4 signaling, it is interesting to note that *A. baumannii* treatment suppressed airway eosinophilia in both TLR4−/− and wild type mice at a similar magnitude (Figure 8). This result argues against a role for LPS in *A. baumannii*–induced suppression of allergy airway eosinophilia. Instead, it suggests that other components of *A. baumannii* may be more important in suppression of OVA-specific inflammation.

*A. baumannii* infection *per se* induces strong inflammatory responses and *A. baumannii* can cause substantial airway epithelial damage via secretion of enzymes and toxic products leading to oxidative stress and epithelial cell apoptosis [52]. As such, it is possible that the reduction in most cytokines and inflammatory cells assessed in *A. baumannii*-treated allergic mice versus PBS-treated allergic mice could be due to increased epithelial cell death. However, this is unlikely since we, and others, have previously shown that i.n. *A. baumannii* infection at this dose generally increases proinflammatory cytokine and chemokine responses in the lungs [19], [20]. Moreover, we also showed that formalin-killed *A. baumannii*, which causes little or no damage to the pulmonary epithelium, also inhibits airway eosinophilia (Figure 4), making it unlikely that epithelial cell death is responsible for the reduction in most cytokines and inflammatory cells seen in this model.

In conclusion, administration of *A. baumannii* to mice that had already been sensitized to OVA is capable of inhibiting airway eosinophilia and associated pulmonary pathology. Our results further support the observed role of microbes and their products on the development/outcome of the pathogenesis of allergic asthma. Future studies to examine the long term effect of treatment, the use of inactivated bacterial cells, and particularly the identification of effective bacterial component(s) should be undertaken to explore *A. baumannii* as a potential immunomodulator for the treatment of human allergic asthma.
